# Cerebrospinal fluid MTBR243 tau correlates with [18F] GTP1 Tau PET and the two biomarkers show similar correlations with clinical measures

**DOI:** 10.1002/alz70861_108306

**Published:** 2025-12-23

**Authors:** Philip B. Verghese, Matthew R. Meyer, Shin‐Cheng Tzeng, Christina Rabe, Julie Lee, Anne Biever, Emilia Calderon, Edmond Teng, Cecilia Monteiro, Felix L Yeh, Kaleb Patania, Venky Venkatesh, Tim West, Tobias Bittner, Joel B. Braunstein

**Affiliations:** ^1^ C2N Diagnostics LLC, St. Louis, MO USA; ^2^ C2N Diagnostics, LLC, Saint Louis, MO USA; ^3^ Genentech, Inc., South San Francisco, CA USA; ^4^ Genentech, South San Francisco, CA USA; ^5^ F. Hoffmann‐LaRoche AG, Basel Switzerland and Genentech Inc. a member of the Roche Group, South San Francisco, CA USA; ^6^ C2N Diagnostics, LLC, St. Louis, MO USA

## Abstract

**Background:**

Tau tangle neuropathology can be visualized in living individuals using radioligands that bind specifically to aggregated tau. MTBR243 is a biomarker that has been found to be strongly associated with the presence of tau tangles in the brain. In this analysis we compared MTBR243 measured in CSF to Tau PET and clinical variables.

**Method:**

MTBR243 was analyzed in 200 baseline CSF samples from study participants from two clinical trials in early and mild‐to‐moderate Alzheimer’s Disease (AD) (Tauriel, NCT03289143 and Lauriet, NCT03828747), including 182 participants who also had Tau PET data using [^18^F]GTP1 tau PET tracer. All subjects were amyloid positive per study inclusion criteria. Tau PET results were analyzed as continuous variable using the Temporal Meta ROI SUVR and dichotomized as low/high based on Temporal Meta ROI SUVR of 1.33. Clinical measures at baseline included CDR‐SB, ADAS‐Cog11, ADCS‐ADL, and MMSE.

**Result:**

The CSF MTBR243 concentration had high correlation with Tau PET SUVR (Spearman’s ρ = 0.69, 95% CI 0.60‐0.76). 69.8% were classified as high Tau PET in the participants who were analyzed for MTBR243 in CSF. The AUC‐ROC of CSF MTBR243 for identifying Tau PET positivity was 0.89 (95% CI: 0.84‐0.94). We found that both Tau PET and CSF MTBR243 correlated with all 4 clinical measures at baseline as well as change from baseline. The correlations for the two biomarkers with clinical measures were very similar.

**Conclusion:**

CSF MTBR243 correlates well with Tau PET in this amyloid positive population and discriminates well between low and high Tau PET patients. Tau PET and CSF MTBR243 have similar correlations to clinical variables, suggesting that CSF MTBR243 could be a suitable candidate as a fluid surrogate biomarker for Tau PET.

